# A high diversity of mechanisms endows ALS-inhibiting herbicide resistance in the invasive common ragweed (*Ambrosia artemisiifolia* L.)

**DOI:** 10.1038/s41598-021-99306-9

**Published:** 2021-10-07

**Authors:** Ingvild Loubet, Laëtitia Caddoux, Séverine Fontaine, Séverine Michel, Fanny Pernin, Benoit Barrès, Valérie Le Corre, Christophe Délye

**Affiliations:** 1 UMR Agroécologie, INRAE, Dijon, France; 2 USC CASPER, Anses, INRAE, Université de Lyon, Lyon, France

**Keywords:** Natural variation in plants, Plant evolution, Invasive species

## Abstract

*Ambrosia artemisiifolia* L. (common ragweed) is a globally invasive, allergenic, troublesome arable weed. ALS-inhibiting herbicides are broadly used in Europe to control ragweed in agricultural fields. Recently, ineffective treatments were reported in France. Target site resistance (TSR), the only resistance mechanism described so far for ragweed, was sought using high-throughput genotyping-by-sequencing in 213 field populations randomly sampled based on ragweed presence. Additionally, non-target site resistance (NTSR) was sought and its prevalence compared with that of TSR in 43 additional field populations where ALS inhibitor failure was reported, using herbicide sensitivity bioassay coupled with ALS gene Sanger sequencing. Resistance was identified in 46 populations and multiple, independent resistance evolution demonstrated across France. We revealed an unsuspected diversity of ALS alleles underlying resistance (9 amino-acid substitutions involved in TSR detected across 24 populations). Remarkably, NTSR was ragweed major type of resistance to ALS inhibitors. NTSR was present in 70.5% of the resistant plants and 74.1% of the fields harbouring resistance. A variety of NTSR mechanisms endowing different resistance patterns evolved across populations. Our study provides novel data on ragweed resistance to herbicides, and emphasises that local resistance management is as important as mitigating gene flow from populations where resistance has arisen.

## Introduction

Among crop pests, weeds are the leading causes for row crop yield losses in agriculture^1^. The use of synthetic herbicides to control weeds since the middle of the twentieth century contributed to increase global crop yields. However, following the intensive use of herbicides to control weeds, a number of weed species evolved mechanisms allowing them to survive and successfully reproduce in treated fields. Herbicide resistance can be defined as the natural, inheritable ability of mutant weed genotypes to survive herbicide concentrations that kill or inhibit the development of wild-type genotypes of the same species (sensitive genotypes), and/or as the outcome of the adaptive evolution of weeds as a result of selection for reduced sensitivity under herbicide selective pressure^2^. The efficacy of herbicides is now threatened by the widespread evolution of resistance. To date, resistance has been reported in 263 weed species worldwide and to 23 of the 26 known herbicides modes of action^3^. The mechanisms underlying herbicide resistance can be classified into two main categories: target site resistance (TSR) and non-target site resistance (NTSR)^4,5^. TSR can be caused by mutation(s) at the gene encoding the target protein of the herbicide, resulting in a modification of the protein structure that reduces herbicide binding. TSR can also be due to an increase in the amount of the herbicide target (gene amplification and/or over-expression). TSR is generally under monogenic control, although there are a few exceptions^5^. By contrast, NTSR is endowed by quantitative and/or qualitative change(s) in the weed secondary metabolism that leads to a reduction in the quantity of active herbicide molecules reaching their target site (e.g. excretion, sequestration, exacerbated metabolism). NTSR is generally considered a complex, polygenic adaptation to herbicide selective pressure that involves large genes families^4,5^.

A diversity of mechanisms is involved in TSR or in NTSR. The amino-acid changes involved in TSR must allow the mutant herbicide target to retain its physiological activity. Thus, a relatively limited set of amino-acid replacements is observed across weed species for a given herbicide target^3,6^. Furthermore, different amino-acid substitutions at a given herbicide target are not always functionally redundant and can confer resistance to different sets of herbicide molecules acting at this target^7^. NTSR is most often a quantitative trait. It is considered to evolve gradually from standing genetic variation by the accumulation of small-effect alleles^4^. Due to this quantitative nature, it is likely that a number of allelic combinations can cause NSTR to a given herbicide. On the other hand, it has been demonstrated that one given NSTR mechanism can cause resistance to herbicides with different targets^8^. At the level of one individual weed plant, herbicide resistance can be caused by one or by several TSR and/or NTSR alleles. As a diversity of such alleles exist, the evolution of herbicide resistance in weed species is most often a complex process, and the evolutionary trajectory of a weed population will depend on the resistance mechanism(s) selected. Furthermore, multiple, independent evolution across populations is likely^9,10^. Yet, few studies so far have explored the diversity of resistance mechanisms within a species on a large geographical scale^4^.

Common ragweed (*Ambrosia artemisiifolia* L.) is one of the most troublesome weeds worldwide. This annual plant from the *Asteracea* family is native from North America^11^, but has spread worldwide, mostly by human mediated seed dispersal^12^. Common ragweed is currently considered as a major weed in agricultural fields, competing with crops for light, water and soil nutrients. It particularly impacts summer crops such as maize, soybean and sunflower^13,14^. Moreover, common ragweed is also a major concern for public health. Its highly allergenic pollen is one of the most important cause of hay fever globally^15^. In agricultural fields, common ragweed is mainly controlled by herbicide applications. As a result, resistance to herbicides targeting photosystem II, protoporphyrinogen oxidase, 5-enolpyruvylshikimate-3-phosphate synthase and/or acetolactate-synthase (ALS) has been reported in this species in its native range (the United States and Canada^3^).

Common ragweed has been introduced in France in 1863^16^. It is currently a growing concern in agriculture, with especially high levels of infestation in sunflower and in soybean. Sunflower cultivars resistant to the ALS-inhibiting herbicides tribenuron or imazamox have been marketed to control weeds difficult to control in “conventional” sunflower cultivars, including ragweed^17^. Imazamox is also used for common ragweed control in soybean. Both herbicides provide efficient ragweed control. Yet, a decrease in their efficacy has recently been reported in highly infested areas, arising the suspicion of the occurrence of resistance to ALS inhibitors. TSR is a frequent and widespread cause of resistance to ALS inhibitors, especially in broadleaf weeds^18,19^. Indeed, TSR to ALS inhibitors endowed by an ALS allele carrying a Trp574Leu substitution observed in the USA is the only mechanism of resistance identified to date in common ragweed^20–22^.

Managing or mitigating herbicide resistance requires understanding its modalities of evolution, and identifying the mechanisms involved. Here, we investigated the occurrence of resistance to ALS inhibitors in French common ragweed populations. In a first step, we used a high-throughput genotyping-by-sequencing approach (Pool-seq) to seek TSR to ALS inhibitors in a massive and national-scale sampling of ragweed populations. In a second step, we investigated the presence of resistance, whatever the underlying mechanism, using herbicide sensitivity bioassays, and compared the respective prevalence of TSR and NTSR mechanisms. The cross-resistance pattern associated to the two major TSR ALS alleles identified in common ragweed was established. In addition, we examined the patterns of resistance evolution among populations.

## Results

### Detection of nine amino-acid substitutions at the ALS gene in French common ragweed populations

Overall, nine nucleotide substitutions causing nine amino-acid changes within the ALS protein were detected in the two ragweed population samplings analysed in this study. Three substitutions occurred at codon 197, two at codon 205, two at codon 574, one at codon 376 and one at codon 578.

**Figure 1:**
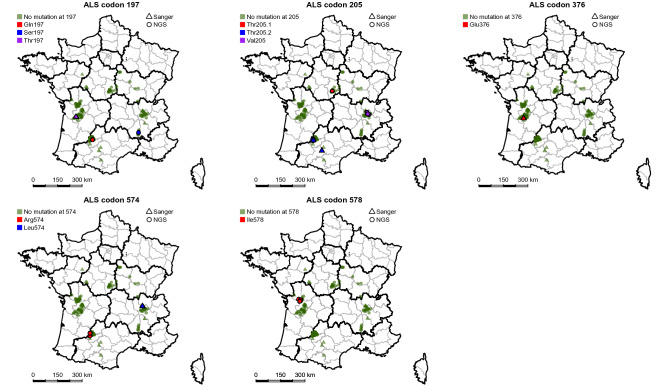
Geographical distribution of the nine mutant ALS alleles identified in this study among common ragweed populations. The results obtained by Pool-seq analysis of the random sampling of 213 populations and by Sanger sequencing of resistant plants from the targeted sampling of 43 additional populations are illustrated together. Green dots, populations where no allele mutant at the considered codon was detected. Coloured dots, populations where mutant ALS alleles were detected by pool-seq. Coloured triangles, populations where mutant ALS alleles were detected by Sanger sequencing. Maps generated using R software (v4.1.0, packages rgdal, rgeos and mapplots).

A random sampling including a total of 213 common ragweed populations collected in areas in France where ragweed is frequently observed in agricultural fields was analysed using Illumina Pool-seq. We detected five amino-acid substitutions at a total of four codons involved or potentially involved in TSR to ALS inhibitors in 15 populations distributed among all sampled regions (Table 1; Fig. 1). Two different substitutions at codon 197 (Pro197Gln and Pro197Ser) were each detected in one population, in a frequency of 1.0 and 2.4% respectively (Table 1). A Trp574Arg substitution was detected in low frequencies (1.6% and 2.1%) in two neighbouring populations. An Ala205Thr substitution was detected in eight populations in frequencies ranging from 1.2 to 11.5%. Seven of these populations formed a geographical cluster. Last, a Phe578Ile substitution was detected in three clustered populations in frequencies ranging from 1.1 to 4.9%. Overall, the frequencies of the mutant ALS alleles in each population were low, with values < 5% in 12 of the 15 populations where such alleles were detected, and a maximum value of 11.5%.

**Table 1:** Mutant ALS alleles detected by Illumina Pool-seq and their estimated within-population frequencies in a random sampling of 213 populations.

Population code	Mutant ALS allele detected	Mutant ALS allele frequency (%)
OCC20	Gln197	1.0
ARA9	Ser197	2.4
OCC14	Thr205	11.5
OCC16	Thr205	5.6
OCC17	Thr205	5.7
OCC21	Thr205	2.0
OCC22	Thr205	1.2
OCC23	Thr205	1.7
OCC24	Thr205	1.1
CVL6	Thr205	4.1
OCC18	Arg574	2.1
OCC19	Arg574	1.6
NAQ10	Ile578	1.1
NAQ11	Ile578	1.4
NAQ12	Ile578	4.9

Only populations where mutant alleles were detected are listed in this table.

The random sampling was completed by a targeted sampling including 43 additional common ragweed populations collected in fields where treatment failures had been reported, and therefore occurrence of resistance could be suspected. All populations were subjected to ALS inhibitor sensitivity bioassays. Sanger sequencing of the ALS gene in the resistant plants identified by these bioassays detected a total of five amino-acid substitutions at four codons involved in TSR to ALS inhibitors (Table 2; Fig. 1). The Ala205Thr substitution detected in the random survey was also found in a geographical cluster of three populations. Four additional substitutions were identified in six populations. An Ala205Val substitution was detected in a geographical cluster of three populations. A Pro197Thr, an Asp376Glu and a Trp574Leu substitution were each detected in one population.

**Table 2:** Occurrence and frequencies of imazamox-resistant plants and of tribenuron-resistant plants in the 31 populations from the targeted sampling (43 populations in total) where at least one resistant plant was detected by herbicide sensitivity bioassays.

Population code	Imazamox				Tribenuron			
	% Resistant plants	% Resistant plants sequenced	Mutation detected	% Mutant, resistant plants	% Resistant plants	% Resistant plants sequenced	Mutation detected	% Mutant, resistant plants
ARA2	10	100	0		0			
ARA3	3	100	Leu574	100	NA			
NAQ4	3	100	0		0			
OCC4	3	100	0		0			
OCC5	3	100	Thr205	100	0			
ARA4	0				15	50	0	
CVL2	0				8	100	0	
CVL3	0				5	50	0	
CVL4	0				8	33	0	
CVL5	0				13	100	0	
NAQ10	0				10	100	Thr197	25
NAQ5	0				5	50	0	
NAQ6	0				16	100	0	
NAQ7	0				15	100	0	
NAQ8	0				43	100	0	
NAQ9	0				18	71	0	
OCC6	0				5	100	0	
OCC7	0				3	100	0	
OCC8	0				3	0	NA	
OCC9	NA				25	0	NA	
ARA5	70	20	Val205	100	70	25	Val205	100
ARA6	5	100	Val205	100	33	77	Val205	33
ARA7	65	20	Val205	100	58	9	Val205	100
ARA8	3	100	0		15	100	0	
NAQ11	3	100	0		03	100	0	
NAQ12	3	100	0		10	100	0	
NAQ13	3	100	Glu376	100	3	0	NA	
OCC10	16	100	Thr205	100	5	0	NA	
OCC11	3	100	0		5	100	Thr205	50
OCC12	5	100	0		5	100	0	
OCC13	5	100	0		10	100	0	

Mutant ALS alleles were sought in resistant plants by Sanger sequencing. Populations are grouped according to the presence of resistance to imazamox only, to tribenuron only, or to both herbicides.

NA not assayed.

### Phylogenetic analysis of mutant ALS alleles

**Figure 2:**
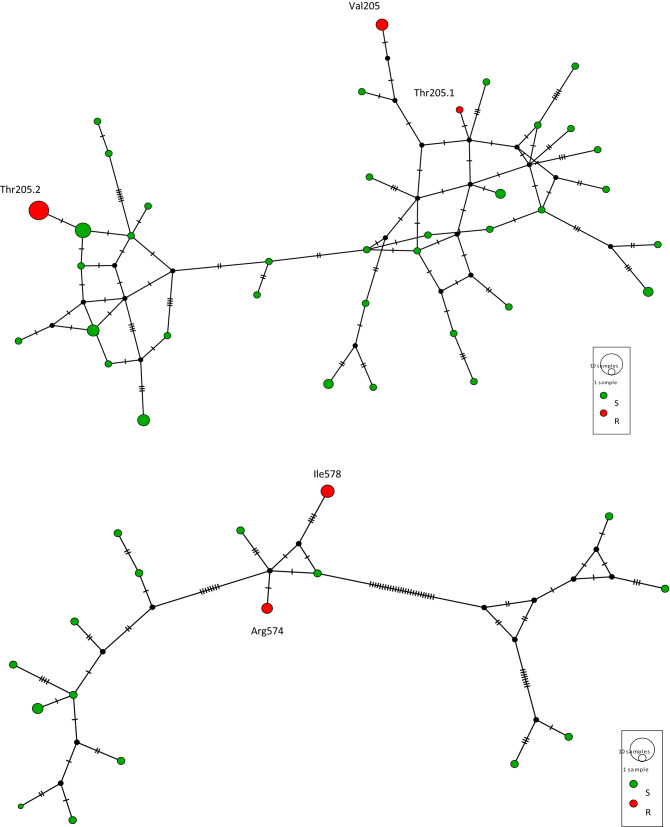
Haplotype network of the ALS haplotypes carrying a mutation at codon 205 (top), 574 or 578 (bottom) (red discs). Green discs, wild-type haplotypes (not carrying a mutation involved in herbicide resistance).

The DNA sequences of all mutant ALS alleles detected in at least two populations (i.e., Thr205, Val205, Arg574 and Ile578) were aligned and a haplotype network established on the basis of the nucleotide polymorphisms observed (Fig. 2). The Val205, the Arg574 and the Ile578 haplotypes had one single evolutionary origin each. These haplotypes had been detected in populations clustered within relatively small regions (Fig. 1). The maximum distance as the crow flies between two populations in our sampling harbouring these haplotypes were 37 km, 18 km and 15 km for the Val205, Arg574 and Ile578 haplotype, respectively. Two different evolutionary origins corresponding to two distinct geographical areas were observed for the Thr205 alleles. Haplotype Thr205.1 was detected in one single population located 327 km apart from the nearest sampled population harbouring a Thr205 allele. The 11 remaining populations all contained a second haplotype (Thr205.2). Of these populations, 10 were clustered within a 10 km radius. The last one was located 83 km apart from the nearest population in the cluster harbouring allele Thr205.2.

### Resistance to the ALS inhibitors tribenuron and imazamox in common ragweed populations

**Figure 3:**
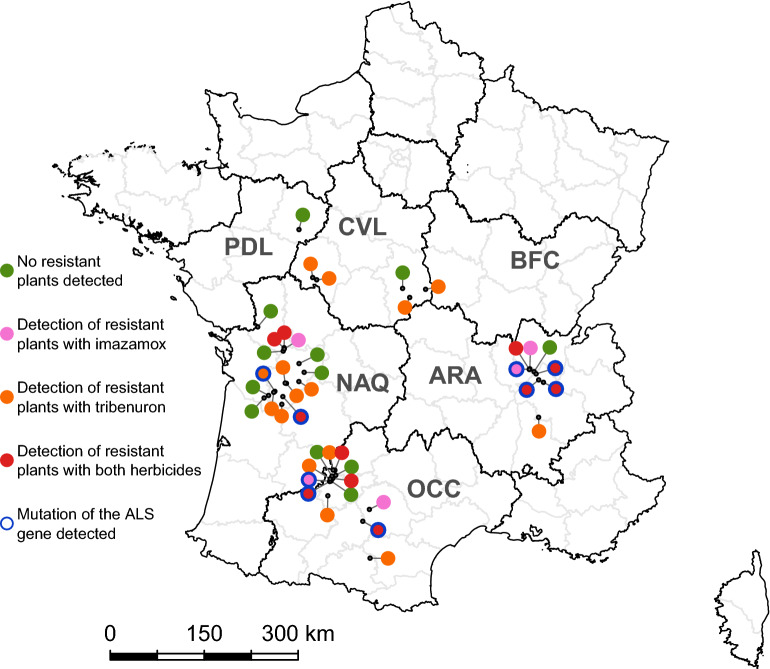
Occurrence of resistance to imazamox, tribenuron and both herbicides in the targeted sampling of 43 common ragweed populations. The presence or absence of resistant plants is indicated by coloured dots. The small grey dot connected to each coloured dot indicates the precise location of the corresponding population. French Région names are coded as follows: ARA, Auvergne Rhône-Alpes; BFC, Bourgogne Franche-Comté; CVL, Centre Val-de-Loire; NAQ, Nouvelle Aquitaine; OCC, Occitanie; PDL, Pays de la Loire. Map generated using R software (v4.1.0, packages rgdal, rgeos and mapplots).

The 43 populations in the targeted sampling were collected on the basis of a suspicion for resistance and assayed for sensitivity to the herbicides imazamox and tribenuron. Resistant plants were identified with at least one of the two herbicides assayed in 31 of the 43 populations. Only imazamox-resistant plants were detected in five populations (four populations tested with both herbicides and one with imazamox only), in frequencies ranging from 3 to 10% (Table 2). Conversely, only tribenuron-resistant plants were detected in 15 populations (14 tested with both herbicides and one with tribenuron only), in frequencies ranging from 3 to 28% (Table 2). Both imazamox-resistant plants and tribenuron-resistant plants were detected in 11 populations, in frequencies ranging from 3 to 10%. In the remaining 12 populations, no plants resistant to the herbicide(s) assayed were detected (seven populations were tested with both herbicides, four with imazamox only and one with tribenuron only) (Fig. 3).

### Mechanisms underlying resistance: TSR

As detailed above and in Table 2, five mutant ALS alleles were detected in resistant plants in nine out of the 31 populations of the targeted sampling where resistance was observed. The resistance phenotype conferred by the two most commonly detected Val205 and Thr205 alleles was confirmed by conducting a combined genotyping-and-phenotyping approach on four populations. The results are summarised in Table 3. In all four populations tested, all homozygous wild-type plants were sensitive to imazamox and tribenuron. All plants with at least one mutant allele were resistant to both herbicides. Overall, Fisher’s exact test showed a clear association of the genotype at ALS with resistance (*p* value = 1.8 × 10^–61^).

**Table 3:** Genotype at ALS codon 205 and sensitivity to imazamox or tribenuron of common ragweed.

Population code	Genotype	Phenotype			
		Number of imazamox-resistant plants	Number of imazamox-sensitive plants	Number of tribenuron-resistant plants	Number of tribenuron-sensitive plants
ARA5	Ala/Ala205	0	9	0	17
	Val/Val205	9	0	7	0
	Ala/Val205	22	0	16	0
ARA7	Ala/Ala205	0	18	NA	NA
	Val/Val205	7	0	NA	NA
	Ala/Val205	15	0	NA	NA
OCC10-bulk1	Ala/Ala205	0	2	0	6
	Thr/Thr205	18	0	14	0
	Ala/Thr205	20	0	19	0
OCC10-bulk2	Ala/Ala205	0	5	0	9
	Thr/Thr205	11	0	16	0
	Ala/Thr205	23	0	15	0

Forty plants were assayed per population and per herbicide.

*NA* not assayed.

The remaining three mutant ALS alleles observed in the targeted population sampling could not be characterised as thoroughly as the Thr250 and Val205 alleles, because of their low frequencies (Table 2). All these alleles were observed in one single population each (Table 2). The Thr197 allele was observed in the heterozygous state in one single tribenuron-resistant plant in population NAQ10. The Glu376 allele was detected in the heterozygous state in the only plant resistant to imazamox sequenced in population NAQ13. The Leu574 allele was observed in the only imazamox-resistant plant identified in population ARA3. Plants from this population could not be assayed with tribenuron, due to poor seed germination.

### Mechanisms underlying resistance: NTSR

Overall, the ALS gene was sequenced in 122 resistant plants from 31 populations, and mutant ALS alleles were identified in 36 plants from nine populations (Table 2). The 11 other resistant plants in these nine populations did not carry any mutant ALS allele. No resistant plant carrying a mutant ALS allele was detected in the remaining 22 populations where resistance was observed.

To confirm that no TSR mechanism was at play in the resistant plants carrying wild-type ALS alleles, ALS expression was compared between resistant plants and sensitive plants in four populations where no mutant ALS allele was detected. ALS expression was not significantly different between resistant and sensitive plants in any of the four populations analysed (Supplementary Figure S1). TSR due to ALS gene overexpression was thus not the mechanism involved in resistance in these plants (Table 2).

To sum up, the presence of NTSR (that is, occurrence of resistant plants not carrying TSR mechanisms) was established in 23 of the 31 populations where resistance was identified (Table 2): three populations where resistance to imazamox only was detected, 13 populations where resistance to tribenuron only was detected, and seven populations with resistance to both herbicides was detected. Over all populations, NTSR mechanisms were estimated to underly resistance in 70.5% of the resistant ragweed plants (95% confidence interval 0.61–0.78).

## Discussion

This is the first national-scale study investigating the occurrence of herbicide resistance in the major weed common ragweed and elucidating the diversity of mechanisms at the root of this resistance. Our study confirmed for the first time the occurrence of resistance to the ALS inhibitors imazamox and tribenuron in common ragweed in France. Resistance was present in several foci scattered across the regions investigated. Resistance was likely emerging in most of the populations where it was detected, with frequencies of resistant plants observed in bioassays in and around a few percent. However, resistance was much more firmly established in a few locations (33–70% resistant plants in three populations in Auvergne Rhône-Alpes, and 43% in one population in Nouvelle Aquitaine). While these rates do not reach those observed in Canada where resistance to ALS inhibitors is widespread and frequent (81% and 100% of ragweed populations uncontrolled by these herbicides in Quebec and in Ontario, respectively, contain substantial frequencies of resistant plants^23,24^), the emergence of resistance observed in our study could herald the beginning of a broad-scale resistance selection if the use of ALS inhibitors is maintained, or even increased to compensate for the decrease in efficacy caused by resistance. Thus, ragweed resistance to ALS inhibitors in France has now become an issue. In areas where common ragweed is widespread and frequent, most applications of ALS inhibitors specifically targeting this weed have been, and still are, performed in sunflower crops or in soybean crops. Indeed, sunflower cultivars resistant to ALS inhibitors were introduced in France in 2010 primarily to allow ragweed control in this crop. Herbicide-resistant sunflower is often included in short rotations including cereal crops, where ALS inhibitors are also used as a weed control solution, or soybean, where imazamox can be applied. As a result of intensive use of ALS inhibitors against common ragweed, resistance has been detected in the two samplings we performed between 2015 and 2019, i.e., half a dozen years after the release of the herbicide-resistant sunflower cultivars. This observation agrees with data from the literature. ALS inhibitors are the herbicide mode of action to which the highest number of resistance cases have been recorded (166 resistant weed species worldwide evolved resistance to these herbicides, and particularly to imazamox and tribenuron^3^). Moreover, common ragweed belongs to the *Asteraceae* taxonomic family, which ranks second in term of the number of species where resistance evolved (45 species so far^3^). Last, the introduction of herbicide-resistant crops has been reported to contribute to the development of herbicide resistance in weeds^25^. As a case in point, the evolution of common ragweed resistance to glyphosate, a low-resistance-risk herbicide^26^, was associated to the use of glyphosate-resistant soybean in the USA^27^.

So far, resistance to herbicides in common ragweed had only been reported in the species native range, i.e., North-America. Most studies published to date pertain to glyphosate, an herbicide inhibiting 5-enolpyruvylshikimate-3-phosphate synthase. Among the studies reporting resistance to ALS inhibitors in common ragweed, only three investigated the mechanisms involved. The first two studies each identified a Leu574 ALS allele in one population^20,21^. The third study also reported the presence of a Leu574 ALS allele, in ten additional populations^22^. The presence of other TSR allele(s) was suspected in some populations on the basis of ALS enzyme assays, but the amino-acid substitutions involved were not identified. Thus, only TSR caused by Leu574 ALS allele had previously been reported in common ragweed. This is in contrast to our study, where the unprecedented combination of high-throughput TSR diagnosis, herbicide bioassays and ALS Sanger sequencing unravelled an unsuspected diversity of TSR alleles. We detected a total of nine amino-acid substitutions, of which two had never been reported before. The previously reported Leu574 allele was detected at a very low frequency in one single population (Table 2). It was not, and by far, the most frequent or widespread allele identified in French common ragweed populations (Tables 1, 2). Leu574 ALS allele has been characterised in numerous weeds and is known to confer a high level of resistance to most ALS inhibitors^18,28^, including imazamox in common ragweed^22^. A second allele carrying a substitution at the same codon (Trp574Arg) was detected in our work. This allele had only been reported before in the grass *Digitaria sanguinalis* (large crabgrass), where it also conferred a broad resistance pattern^29^. Three alleles carried a substitution at codon 197 (Gln197, Ser197 and Thr197), each previously shown in other species to confer a high resistance to sulfonylureas like tribenuron, and a variable resistance to imidazolinones like imazamox^19^. The same applies to the Glu376 allele identified in our work. Conversely, the Val205 allele was reported to confer a high resistance to imidazolinones and a variable resistance to sulfonylureas^19^. Herein, we demonstrated that this allele confers dominant resistance to both tribenuron and imazamox in common ragweed (Table 3). The last two alleles identified in our survey were both novelties. Characterisation of the resistance endowed by the Thr205 allele yielded results similar to those obtained for the Val205 allele, i.e., dominant resistance to tribenuron and imazamox (Table 3). The last ALS allele identified was a putative TSR allele. It carried a Phe-to-Ile substitution at codon 578. Seven amino-acid substitutions at codon 578 have been studied in yeast or in tobacco laboratory mutants, and shown to alter sensitivity or to confer resistance at least to sulfonylureas herbicides^30,31^. However, ALS alleles carrying a substitution at codon 578 had never been observed before in weed populations from the field. Furthermore, the Phe-to-Ile substitution we identified at position 578 is not among the seven amino-acid substitutions characterised at this codon^30,31^. Herbicide bioassays on plants from the originating fields will be necessary to confirm the novel Ile578 allele is actually a TSR ALS allele, and to characterise its associated resistance phenotype.

The diversity of the ALS alleles detected across common ragweed populations in our French sampling exceeds what had been previously described in most other weed species worldwide. Indeed, up to six variable amino-acid positions were previously recorded in a single species (*Amaranthus retroflexus*), while up to eight amino-acid changes at no more than four positions were described in each of the four species *Apera spica-venti*, *Descurainia sophia*, *Kochia scoparia* and *Schoenoplectus juncoides*^19^. The unprecedented diversity observed in our study is partly attributable to a methodological breakthrough. Indeed, our study is the first broad-scale resistance survey implementing high-throughput genotyping-by-sequencing to detect variants at all known resistance-associated positions within the ALS gene. In the future, applying this method to other pests that have evolved pesticide resistance will undoubtedly reveal more diverse genetic bases for TSR than presently recorded.

Our broad sampling enabled us to detect mutations and resistant plants in very low frequencies, and therefore to detect resistance at its emergence. Previous monitoring studies mostly considered resistance distribution or prevalence at a much more advanced stage^32,33^. In particular, we observed the appearance of TSR clusters, including two independent events of appearance of the Ala205Thr substitution in geographically distant areas. Redundant evolution of TSR by multiple, independent appearances of TSR alleles had been previously observed in other species (e.g.,^34,35^), including species that evolved TSR to ALS inhibitors (e.g., *Papaver rhoeas*^36^ and *Amaranthus tuberculatus*^10^). Our results also suggest that once a TSR haplotype has evolved, its geographical spread in a region is rapid. In the 5–8 years that elapsed between the onset of ALS inhibitor selective pressure caused by herbicide-resistant sunflower release and our sampling, one TSR allele (Thr205.2) spread over a distance of c.a. 150 km (Fig. 1). Wind-borne pollen like that of common ragweed is a vector for herbicide resistance (e.g.,^35,37^). However, long-distance dispersal of common ragweed is mainly mediated by seeds moved by human activity, especially agricultural machinery^38^. It is likely that the dispersion of herbicide resistance in common ragweed over an agricultural production basin is promoted by agricultural activities.

While our work unravelled a surprisingly high diversity of TSR alleles in common ragweed, a major outcome of this study was the unexpected predominance of NTSR mechanisms. Indeed, the majority of common ragweed populations where resistance was detected using bioassay did not harbour TSR alleles (Table 2). Occurrence of TSR by increased ALS expression, be it due to enhanced transcription or ALS gene amplification, was ruled out using ALS expression measurement (Supplementary Figure S1). Our results agree with TSR to ALS inhibitors by increased target content being rare, with only one case reported to date in the literature in the grass weed *Hordeum leporinum*^39^.

NTSR had already been reported in common ragweed to other herbicides modes of action, such as photosystem II inhibitors^40^ and glyphosate^41^ in Canada. Ours is the first report worldwide of NTSR to ALS inhibitors in common ragweed. NTSR to ALS inhibitors had already frequently been reported in grass weeds^7,42–44^, but very seldom in dicotyledonous weeds, with four species concerned so far: *Amaranthus hybridus*^45^, *Sinapis arvensis*^46,47^, *P. rhoeas*^48^ and *Amaranthus palmeri*^49^. Here, we observed that NTSR is largely distributed in French common ragweed populations and can cause alone high resistance frequencies. Resistance patterns varied with the population, and resistance to both herbicides assayed was not systematically associated. Rather, evolution of NTSR to imazamox alone or to tribenuron alone seemed to be the preferred pattern observed. This is consistent with imazamox-resistant sunflower and tribenuron-resistant sunflower being mutually exclusively grown on vast areas, as a result of the different requirements of the respective cultivars and of competition between agricultural cooperatives. This is also consistent with imazamox and tribenuron displaying different chemistries while acting at the same target site. This data combined to our results suggest that the metabolic pathways involved in imazamox or in tribenuron degradation are at least in part different, and that independent events of evolution of NTSR occurred across France.

## Conclusions

In conclusion, our work unravelled an unexpected diversity of herbicide resistance mechanisms in common ragweed in France. Common ragweed is a striking example of multiple, parallel evolution where a diversity of TSR and/or NTSR mechanisms are selected for locally, and combined within populations as well as within individual plants. Common ragweed is a highly genetically variable species. Invasive populations of this weed have especially high levels of genetic diversity due to genetic admixture among different colonization sources^50,51^. This may be a reason why the diversity of resistance mechanisms observed in its invasive range appears greater than reported from its native range. Given the detrimental impact of common ragweed on both agricultural production and human health, it is of high importance to be able to monitor herbicide resistance so as to adapt agricultural practices and keep common ragweed under control. High-throughput resistance diagnosis using massive genotyping-by-sequencing is certainly a tool of choice for this purpose. Yet, implementing such a tool requires genes and mutations at the root of resistance to be identified. While this is the case for TSR, the genetic determinants of NTSR to ALS inhibitors remains unresolved. This is all the more unfortunate that the bulk of resistance in common ragweed seems to be endowed by NTSR mechanisms. There is thus an urgent need to gain a better understanding on the genes and alleles driving common ragweed NTSR to ALS inhibitors.

## Materials and methods

### Ragweed samplings

Two samplings were conducted in this study (Supplementary Figure S2). To check whether TSR to ALS inhibitors was present in French ragweed populations, a total of 213 agricultural fields were randomly sampled in five regions faced at least locally with high levels of common ragweed infestation: Auvergne Rhône-Alpes (ARA), Bourgogne Franche-Comté (BFC), Centre Val de Loire (CVL), Nouvelle Aquitaine (NAQ) and Occitanie (OCC) (Supplementary Table S1). Areas of high ragweed presence were criss-crossed by car during summer, and fields were sampled on the sole basis of the presence of common ragweed, without any a priori information on the efficacy of herbicides. One leaf was collected on each of 50 different plants per field sampled prior to TSR diagnosis (see below). Populations were sampled between 2015 and 2018. Overall, 18 populations were sampled in 2015, 68 in 2016, 112 in 2017 and 15 in 2018.

In a second step, to assess the relative importance of TSR and NTSR in common ragweed resistance to ALS inhibitors in France, a second, targeted sampling was performed (Supplementary Table S1). Seeds were collected from 43 additional fields selected on the basis of farmers having reported a poor ALS-inhibitor-based control of common ragweed. This information was obtained through the channels of the national French monitoring system of the adverse effects of phytosanitary products. The fields sampled had in common a history of ALS-inhibiting herbicide applications against common ragweed, but the detail of this history could not be obtained. They were mostly cultivated with sunflower cultivars resistant to ALS inhibitors, with soybean or with maize. Tribenuron (a sulfonylurea) and imazamox (an imadazolinone) were largely the main ALS-inhibiting herbicides applied in these fields. In every field, ripe seeds were collected as a bulk from at least 30 different common ragweed plants. Seeds batches were stored at room temperature for at least 3 months to allow post-ripening before being used for resistance analysis. Populations were sampled between 2017 and 2019. Overall, 28 populations were sampled in 2017, 8 in 2018 and 7 in 2019.

This study complies with the French legislation and institutional guidelines. Permission for collection of ragweed leaves and seeds were obtained in the framework of the monitoring system of the adverse effects of phytosanitary products instituted by the French law of October 13, 2014 on the future of agriculture.

### TSR detection using Illumina high-throughput sequencing technology

The 213 randomly sampled common ragweed populations were analysed following the protocol described in a previous study^52^. Briefly, discs were punched out of the 50 leaves representing each population, pooled in one tube per population and ground in liquid nitrogen. DNA extraction was performed following the rapid DNA extraction procedure described in a previous study^53^. DNA extracts were stored at − 20 °C. PCR were performed on diluted DNA extracts (1/10) and mixes prepared as described^52^. The PCR programs consisted of 3 min at 95 °C, followed by 37 cycles of 5 s at 95 °C, 10 s at 60 °C and 30 s at 72 °C. Three regions of the ALS gene were amplified separately from each DNA extract with the primer pairs including overhang adapter sequences at their 5′ end described elsewhere^52^. These regions carried the eight codons involved in TSR to ALS inhibitors (codons 122, 197, 205, 376, 377, 574, 653 and 654, as standardised after *Arabidopsis* ALS sequence)^28^ and seven additional codons potentially involved in TSR (codons 121, 124, 196, 199, 375, 571 and 578)^54^. All three amplicons were subsequently pooled in an equimolar mix for each population. Each of the 213 resulting mixes was tagged with one specific index. Tagged amplicons were purified and loaded onto one Illumina MiSeq V3 cartridge according to the manufacturer instructions. The 250-nucleotide pair-end sequences passing Illumina standard quality controls were assigned to their population of origin on the basis of the population-specific indexes. Nucleotide changes causing amino-acid substitutions at the 15 ALS codons targeted were sought in each population using the R-based pipeline described elsewhere^52^. Briefly, the reads were aligned to a reference sequence of common ragweed ALS (GenBank accession KX870184). Variant calling was performed at each of the nucleotide positions in the 15 targeted codons where nucleotide changes were predicted to cause amino-acid substitutions. To account for Illumina sequencing errors, we applied a frequency threshold of 1% for the detection of any such change. Then, frequencies of single-nucleotide substitutions at the 15 ALS codons implicated in herbicide resistance were calculated in every pool of 50 plants.

### Resistance detection using herbicide sensitivity bioassays

Seeds were stratified at 4 °C during one month to break dormancy and transferred to a growth chamber for germination (photoperiod of 16 h, 20 °C/15 °C day/night). After germination, seedlings at the cotyledon stage were transplanted into plastic trays (17 × 12.5 × 5.5 cm) filled with 60% silt-loam soil, 15% sand, 15% perlite and 10% peat by volume and placed in a greenhouse (20 °C/15 °C day/night with a 16 h photoperiod). Plants were watered as needed until the two-to-four leaf stage, when they were subjected to herbicide treatment. Each tray contained 20 seedlings. Two trays per population (40 seedlings) were sprayed per herbicide dose, and one tray per population (20 seedlings) was sprayed with water and served as the untreated control. Herbicides were applied using a custom-built, single-nozzle (nozzle AXI 110°06; Albuz, France) sprayer delivering herbicide in 300 L ha^−1^ water at 400 kPa under a temperature of 23 °C and a relative humidity of 80%. The two major ALS inhibitors used to control common ragweed in France were sprayed as commercial formulations at their respective, maximum recommended French field rate. The commercial herbicides used were Express SX^®^ (FMC, 50% w/w tribenuron, field rate of 30 g tribenuron ha^−1^) with 1 L ha^−1^ surfactant (Actirob B^®^, Bayer CropScience), and Pulsar 40^®^ (BASF, 40 g L^−1^ imazamox, field rate of 50 g imazamox ha^−1^) with 1.25 L ha^−1^ surfactant (Dash^®^, BASF).

Every spraying experiment included one batch of 20 plants from the reference population P08 (100% plants sensitive to any herbicide used to control ragweed and applied at the recommended French field rate) for each of the herbicides and herbicide rates assayed, as a check for herbicide application efficiency. Phenotype rating based on visual injury was performed four weeks after treatment, when all plants from the reference population were clearly dead and plant phenotype rating could be performed without ambiguity. Preliminary experiments demonstrated that plants with visually estimated biomass ranging from unaffected by herbicide application (identical to the untreated control plants, 100%) down to ≥ 50% of that of the untreated control plants ultimately recovered and were able to produce viable seeds. They were thus considered resistant. Plants injured and with a visually estimated biomass < 50% of that of the untreated control plants ultimately died. These plants and the plants dead at the time of rating were thus considered sensitive. After phenotype rating, one leaf fragment was collected on plants rated resistant and stored at − 20 °C prior to ALS gene Sanger sequencing.

### ALS gene Sanger sequencing and genotyping

DNA was extracted as before from the leaf fragments collected on the resistant plants. The ALS gene was amplified as two overlapping fragments encompassing the whole ALS coding sequence. A 1237-nucleotide amplicon was amplified using primers ALA5 (5′-IAICACACTCATTCAACAATGGC-3′ where I indicates an inosine base) and ALA4R (5′-CAAAATCTCGTTAAGCCCCTGTAAC-3′), and a 667-nucleotide amplicon was amplified using primers ALA6 (5′-GGGTCGGGCAGCATCAGATG-3′) and ALA1R (5′-ACTGATCCATAACATTCATAACCAG-3′). PCR mixes were as before. For both PCRs, cycling programs consisted of 95 °C 3 min followed by 40 cycles of 95 °C 10 s, 60 °C 15 s and 72 °C 90 s. Both fragments were amplified from each individual plant in three independent PCRs and subsequently sequenced on both strands using Sanger sequencing. The 15 codons screened by Illumina genotyping-by-sequencing where visually checked from Sanger chromatograms using the BioEdit software.

### Haplotype networks for mutant ALS alleles

All sequence reads obtained either from Sanger sequencing or from Illumina sequencing were aligned and compared within and among the populations where they had been detected. Common ragweed is a diploid species^15^, and some of the plants carrying a mutant allele were heterozygous. In these cases, the Sanger sequence of the heterozygous plant was aligned with homozygous sequences obtained from the same population, and its mutant haplotype was deduced from the alignment. When mutant alleles had been identified by Illumina sequencing, the paired reads encompassing the mutant codon were merged before alignment. To account for Illumina sequencing errors, only polymorphisms present at a frequency > 1% of the total of the reads were considered. Haplotype networks were inferred with PopART^55^ using the Median joining method.

### Cross-resistance pattern of the two major ALS TSR alleles to imazamox and tribenuron

The most common and widespread TSR alleles identified carried an Ala205Val or an Ala205Thr substitution. To characterize their cross-resistance pattern to imazamox and tribenuron, common ragweed populations where these alleles were present in high frequencies were required. This criterion was fulfilled for the Val205 allele (populations ARA5 and ARA7, Table 2) but not for the Thr205 allele that was present with a maximum frequency of 16% in the populations sampled (population OCC10, Table 2). To overcome this issue, two populations segregating the Thr205 allele were obtained by separately bulk-crossing two batches of a dozen plants each from population OCC10. The presence of the Thr205 allele at the homozygous or heterozygous state in each of the parental plants had been ascertained beforehand by dCAPS genotyping (see below). The two field populations and the two bulked segregating progenies were subjected to herbicide sensitivity bioassays. To correlate plant phenotype and genotype at the ALS gene, plants were individually identified from their position in the growing tray (40 plants per population and per herbicide), and a piece of young leaf tissue was collected 24 h before herbicide application and stored at − 20 °C until ALS genotyping. Plants were individually genotyped at ALS codon 205 using the derived Cleaved Amplified Polymorphism Sequence technique (dCAPS^56^) and applying previously described guidelines^57^. The dCAPS primer ALAMwoI (5′-GCTATAACTGGTCAAGTTCCAAGAAGAATGATTGCAACAG-3′) introduces a *Mwo*I restriction site in ALS sequences encoding an alanine residue at codon 205 (wild-type). Any substitution at any of the 2 first nucleotides in codon 205 shall remove this restriction site (substitutions at the third nucleotide in codon 205 are silent). This primer was used in PCR with primer ALA7R (5′-GACAAATAACCCGGTAACCTCATAGG-3′). PCR mixes were as before. Cycling program consisted of 95 °C 5 min followed by 37 cycles of 95 °C 5 s, 65 °C 10 s and 72 °C 30 s. Digestion was performed at 37 °C for 3 h using 5 µL digestion mix of *Mwo*I isoschizomer *Hpy*F10VI (Thermo Fisher Scientific, Waltham, MA, USA) and 5 µL of the PCR mix. dCAPS patterns were visualised by electrophoresis on 3% agarose gel in 0.5× TBE Buffer. The genotype of each plant was associated to its phenotype after herbicide bioassay rating.

### Relative ALS gene expression between sensitive and resistant plants

A number of plants surviving ALS inhibitor application did not carry TSR allele(s). To investigate the possibility of these plants being resistant because of an increased content in ALS, the relative expression level of this gene was compared between resistant and sensitive plants in four populations. These populations were selected for their relatively high frequencies of plants resistant to at least one of the herbicides tested (imazamox for population ARA2, tribenuron for population NAQ8, and both herbicides for populations ARA8 and OCC13). Ninety-six individual plants per population were grown in 96-well trays to individualise each seedling, as well as eight plants from the reference population P08. At the four-leaf stage, the apical bud of each plant was harvested in liquid nitrogen in 2 mL tubes containing steel beads and stored at − 80 °C. Twenty-four hours after bud collection, plants were sprayed with imazamox or tribenuron, as described previously. Plant phenotype was rated as previously.

Total RNA extraction of apical buds was performed using the Direct-zol™ RNA MiniPrep (Zymo research) kit and following the manufacturer’s instructions. Total RNA concentration and quality were assessed using a NanoDrop spectrophotometer (LABTECH, Luton, UK). cDNA was synthesised from 1 µg total RNA of quality samples using the Quantitect^®^ Reverse Transcription Kit (Qiagen, Courtaboeuf, France). qPCR was performed from eightfold diluted cDNA samples using the Viaa7 system (Applied Biosystem). A seven-point standard curve based on a two-fold dilution series (from 1:2 to 1:128) of a pool of cDNAs from resistant and sensitive plants was included in the experiment as a control for the efficiency of the qPCR. The relative expression level of the ALS gene was compared between resistant and sensitive plants using the 2ΔΔC_t_ method^58^ after normalisation with three reference genes validated beforehand following the recommendations of^59^ (Supplementary Table S2). The plants from the reference population P08 were used as the reference sample for ALS expression normalisation. ALS relative expression levels of resistant and sensitive plants were compared within each of the four populations using a non-parametric Wilcoxon test.

## Supplementary Information

Supplementary Information 1.

Supplementary Information 2.

## References

[1] Oerke E-C (2006). Crop losses to pests. J. Agric. Sci..

[2] R4P Network. Trends and challenges in pesticide resistance detection. *Trends Plant Sci.* **21**, 834–853 (2016). 10.1016/j.tplants.2016.06.006 27475253

[3] Heap, I. M. The international herbicide-resistant weed database. http://www.weedscience.org/Home.aspx (2021).

[4] Délye C, Jasieniuk M, Le Corre V (2013). Deciphering the evolution of herbicide resistance in weeds. Trends Genet..

[5] Gaines TA (2020). Mechanisms of evolved herbicide resistance. J. Biol. Chem..

[6] Murphy BP, Tranel PJ (2019). Target-site mutations conferring herbicide resistance. Plants.

[7] Beckie HJ, Tardif FJ (2012). Herbicide cross resistance in weeds. Crop Prot..

[8] Han H (2021). Cytochrome P450 CYP81A10v7 in *Lolium rigidum* confers metabolic resistance to herbicides across at least five modes of action. Plant J..

[9] Kreiner JM (2019). Multiple modes of convergent adaptation in the spread of glyphosate-resistant *Amaranthus tuberculatus*. Proc. Natl. Acad. Sci..

[10] Milani A (2021). Population structure and evolution of resistance to acetolactate synthase (ALS)-inhibitors in *Amaranthus tuberculatus *in Italy. Pest Manag. Sci..

[11] Clements DR (2004). Adaptability of plants invading North American cropland. Agric. Ecosyst. Environ..

[12] Essl F (2015). Biological flora of the British Isles: *Ambrosia artemisiifolia*. J. Ecol..

[13] Cowbrough MJ, Brown RB, Tardif FJ (2003). Impact of common ragweed (*Ambrosia artemisiifolia*) aggregation on economic thresholds in soybean. Weed Sci..

[14] Swinton SM, Buhler DD, Forcella F, Gunsolus JL, King RP (1994). Estimation of crop yield loss due to interference by multiple weed species. Weed Sci..

[15] Bassett, I. J. & Crompton, C. W. The biology of Canadian weeds: *Ambrosia artemisiifolia* L. and *A*. *psilostachya* DC. *Can. J. Plant Sci.* **55**, 463–476 (1975).

[16] Chauvel B, Dessaint F, Cardinal-Legrand C, Bretagnolle F (2006). The historical spread of *Ambrosia artemisiifolia* L. France from herbarium records. J. Biogeogr..

[17] Sala CA, Bulos M, Altieri E, Ramos ML (2012). Genetics and breeding of herbicide tolerance in sunflower. Helia.

[18] Yu Q, Powles SB (2014). Resistance to AHAS inhibitor herbicides: Current understanding. Pest Manag. Sci..

[19] Tranel, P. J., Wright, T. R. & Heap, I. M. ALS mutations from resistant weeds. http://www.weedscience.com (2021).

[20] Patzoldt WL, Tranel PJ, Alexander AL, Schmitzer PR (2001). A common ragweed population resistant to cloransulam-methyl. Weed Sci..

[21] Rousonelos SL, Lee RM, Moreira MS, VanGessel MJ, Tranel PJ (2012). Characterization of a common ragweed (*Ambrosia artemisiifolia*) population resistant to ALS- and PPO-inhibiting herbicides. Weed Sci..

[22] Zheng D, Patzoldt WL, Tranel PJ (2005). Association of the W574L ALS substitution with resistance to cloransulam and imazamox in common ragweed (*Ambrosia artemisiifolia*). Weed Sci..

[23] Van Wely, A. C. *et al.* Glyphosate and acetolactate synthase inhibitor resistant common ragweed (*Ambrosia artemisiifolia* L.) in southwestern Ontario. *Can. J. Plant Sci.* **95**, 335–338 (2015)

[24] Marsan-Pelletier, F., Vanasse, A., Simard, M.-J. & Cuerrier, M.-E. Survey of imazethapyr-resistant common ragweed (*Ambrosia artemisiifolia* L.) in Quebec. *Phytoprotection* **99**, 36–44 (2019).

[25] Owen MD, Zelaya IA (2005). Herbicide-resistant crops and weed resistance to herbicides. Pest Manag. Sci..

[26] Duke SO, Powles SB (2008). Glyphosate: A once-in-a-century herbicide. Pest Manag. Sci..

[27] Barnes, E. R., Knezevic, S. Z., Sikkema, P. H., Lindquist, J. L. & Jhala, A. J. Control of glyphosate-resistant common ragweed (*Ambrosia artemisiifolia* L.) in glufosinate-resistant soybean [*Glycine max* (L.) Merr]. *Front. Plant Sci.* **8**, 1455 (2017). 10.3389/fpls.2017.01455 PMC5563374 28868065

[28] Tranel PJ, Wright TR (2002). Resistance of weeds to ALS-inhibiting herbicides: What have we learned?. Weed Sci..

[29] Li J, Li M, Gao X, Fang F (2017). A novel amino acid substitution Trp574Arg in acetolactate synthase (ALS) confers broad resistance to ALS-inhibiting herbicides in crabgrass (*Digitaria sanguinalis*). Pest Manag. Sci..

[30] Duggleby, R. G., Pang, S. S., Yu, H. & Guddat, L. W. Systematic characterization of mutations in yeast acetohydroxyacid synthase. Interpretation of herbicide-resistance data. *Eur. J. Biochem.* **270**, 2895–2904 (2003). 10.1046/j.1432-1033.2003.03671.x 12823560

[31] Jung S-M (2004). Amino acid residues conferring herbicide resistance in tobacco acetohydroxyacid synthase. Biochem. J..

[32] Owen MJ, Walsh MJ, Llewellyn RS, Powles SB (2007). Widespread occurrence of multiple herbicide resistance in Western Australian annual ryegrass (*Lolium rigidum*) populations. Aust. J. Agric. Res..

[33] Owen MJ, Martinez NJ, Powles SB (2014). Multiple herbicide-resistant *Lolium rigidum* (annual ryegrass) now dominates across the Western Australian grain belt. Weed Res..

[34] Délye, C. Nucleotide variability at the acetyl coenzyme A carboxylase gene and the signature of herbicide selection in the grass weed *Alopecurus myosuroides* (Huds.). *Mol. Biol. Evol.* **21**, 884–892 (2004). 10.1093/molbev/msh095 15014166

[35] Délye C, Clément JAJ, Pernin F, Chauvel B, Le Corre V (2010). High gene flow promotes the genetic homogeneity of arable weed populations at the landscape level. Basic Appl. Ecol..

[36] Délye, C., Pernin, F. & Scarabel, L. Evolution and diversity of the mechanisms endowing resistance to herbicides inhibiting acetolactate-synthase (ALS) in corn poppy (*Papaver rhoeas* L.). *Plant Sci.* **180**, 333–342 (2011). 10.1016/j.plantsci.2010.10.007 21421378

[37] Sudheesh M (2015). An analysis of polygenic herbicide resistance evolution and its management based on a population genetics approach. Basic Appl. Ecol..

[38] Bullock, J. M. Assessing and controlling the spread and the effects of common ragweed in Europe. *Report, Contractor: Natural environment research Council UK* (2012).

[39] Yu Q, Nelson JK, Zheng MQ, Jackson J, Powles SB (2007). Molecular characterisation of resistance to ALS-inhibiting herbicides in *Hordeum leporinum* biotypes. Pest Manag. Sci..

[40] Simard M-J, Laforest M, Soufiane B, Benoit DL, Tardif F (2017). Linuron resistant common ragweed (*Ambrosia artemisiifolia*) populations in Quebec carrot fields: presence and distribution of target-site and non-target site resistant biotypes. Can. J. Plant Sci..

[41] Ganie, Z., Jugulam, M., Varanasi, V. & Jhala, A. J. Investigating mechanism of glyphosate resistance in a common ragweed (*Ambrosia artemisiifolia* L.) biotype from Nebraska. *Can. J. Plant Sci.* (2017). 10.1139/CJPS-2017-0036.

[42] Duhoux, A., Carrère, S., Duhoux, A. & Délye, C. Transcriptional markers enable identification of rye-grass (*Lolium* sp.) plants with non-target-site-based resistance to herbicides inhibiting acetolactate-synthase. *Plant Sci.* **257**, 22–36 (2017). 10.1016/j.plantsci.2017.01.009 28224916

[43] Gardin JAC, Gouzy J, Carrère S, Délye C (2015). ALOMYbase, a resource to investigate non-target-site-based resistance to herbicides inhibiting acetolactate-synthase (ALS) in the major grass weed *Alopecurus myosuroides* (black-grass). BMC Genomics.

[44] Torra, J. *et al.* Target-site and non-target-site resistance mechanisms confer multiple and cross- resistance to ALS and ACCase inhibiting herbicides in *Lolium rigidum* from Spain. *Front. Plant Sci.* **12**, 625138 (2021). 10.3389/fpls.2021.625138 PMC7889805 33613607

[45] Manley BS, Hatzios KK, Wilson HP (1999). Absorption, translocation, and metabolism of chlorimuron and nicosulfuron in imidazolinone-resistant and susceptible smooth pigweed (*Amaranthus hybridus*). Weed Technol..

[46] Jeffers GM, O’Donovan JT, Hall LM (1996). Wild mustard (*Brassica kaber*) resistance to ethametsulfuron but not to other herbicides. Weed Technol..

[47] Veldhuis, L. J., Hall, L. M., O’Donovan, J. T., Dyer, W. & Hall, J. C. Metabolism-based resistance of a wild mustard (*Sinapis arvensis* L.) biotype to ethametsulfuron-methyl. *J. Agric. Food Chem.* **48**, 2986–2990 (2000). 10.1021/jf990752g 10898653

[48] Scarabel L, Pernin F, Délye C (2015). Occurrence, genetic control and evolution of non-target-site based resistance to herbicides inhibiting acetolactate synthase (ALS) in the dicot weed *Papaver rhoeas*. Plant Sci..

[49] Nakka S, Thompson CR, Peterson DE, Jugulam M (2017). Target site-based and non-target site based resistance to ALS Inhibitors in Palmer Amaranth (*Amaranthus palmeri*). Weed Sci..

[50] Meyer, L. *et al.* New gSSR and EST-SSR markers reveal high genetic diversity in the invasive plant *Ambrosia artemisiifolia* L. and can be transferred to other invasive *Ambrosia* species. *PLOS ONE* **12**, e0176197 (2017). 10.1371/journal.pone.0176197 PMC5425025 28489870

[51] Van Boheemen LA (2017). Multiple introductions, admixture and bridgehead invasion characterize the introduction history of *Ambrosia artemisiifolia* in Europe and Australia. Mol. Ecol..

[52] Délye C (2020). Harnessing the power of next-generation sequencing technologies to the purpose of high-throughput pesticide resistance diagnosis. Pest Manag. Sci..

[53] Délye C, Matéjicek A, Gasquez J (2002). PCR-based detection of resistance to acetyl-CoA carboxylase-inhibiting herbicides in black-grass (*Alopecurus myosuroides* Huds) and ryegrass (*Lolium rigidum* Gaud). Pest Manag. Sci..

[54] Duggleby RG, McCourt JA, Guddat LW (2008). Structure and mechanism of inhibition of plant acetohydroxyacid synthase. Plant Physiol. Biochem..

[55] Leigh JW, Bryant D (2015). POPART: full-feature software for haplotype network construction. Methods Ecol. Evol..

[56] Neff MM, Neff JD, Chory J, Pepper AE (1998). dCAPS, a simple technique for the genetic analysis of single nucleotide polymorphisms: Experimental applications in *Arabidopsis thaliana* genetics. Plant J..

[57] Délye C, Boucansaud K (2008). A molecular assay for the proactive detection of target site-based resistance to herbicides inhibiting acetolactate synthase in *Alopecurus myosuroides*. Weed Res..

[58] Livak KJ, Schmittgen TD (2001). Analysis of relative gene expression data using real-time quantitative PCR and the 2ddCT method. Methods.

[59] Bustin SA (2009). The MIQE guidelines: minimum information for publication of quantitative real-time PCR experiments. Clin. Chem..

