# Supplementary material for: A high diversity of mechanisms endows ALS-inhibiting herbicide resistance in the invasive common ragweed (Ambrosia artemisiifolia L.)
Source: Sci Rep. 2021 Oct 7;11:19904. doi: 10.1038/s41598-021-99306-9 (PMC8497474; doi:10.1038/s41598-021-99306-9)
Supplement: Supplementary file 1 — Supplementary Information 1. [file 41598_2021_99306_MOESM1_ESM.docx]

**
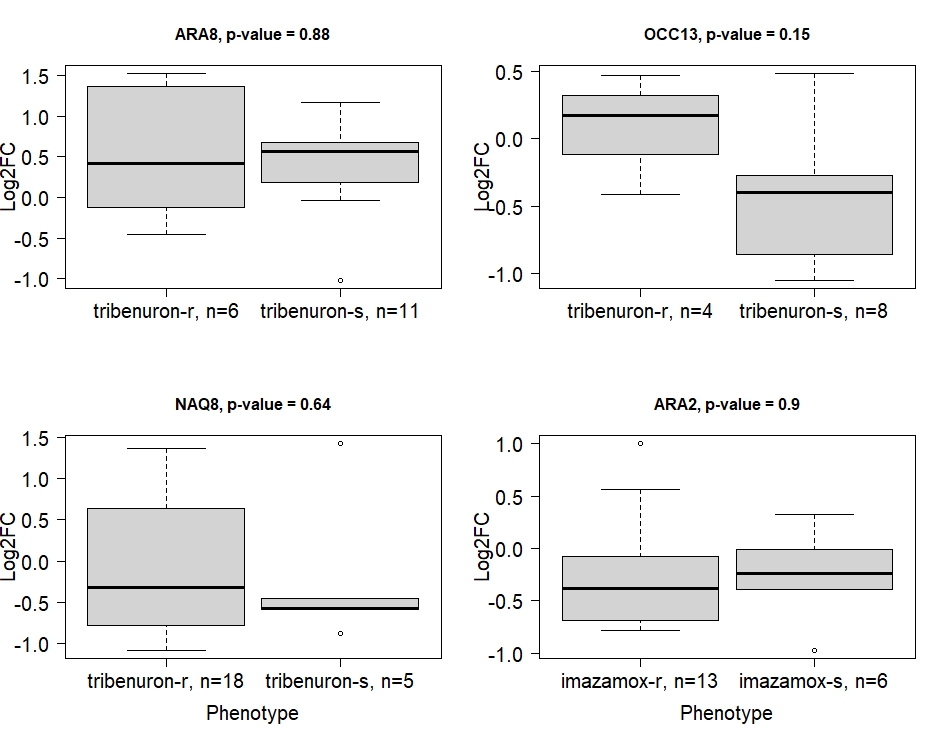
**

**Supplementary Figure 1:** Relative expression level of the ALS gene in resistant and sensitive plants from populations where resistance to imazamox only (ARA2), to tribenuron only (NAQ8) or to both herbicides (OCC13 and ARA8) was observed. P-values are given for the non-parametric Wilcoxon test checking the significance of the difference of relative expression between resistant and sensitive plants in each population.


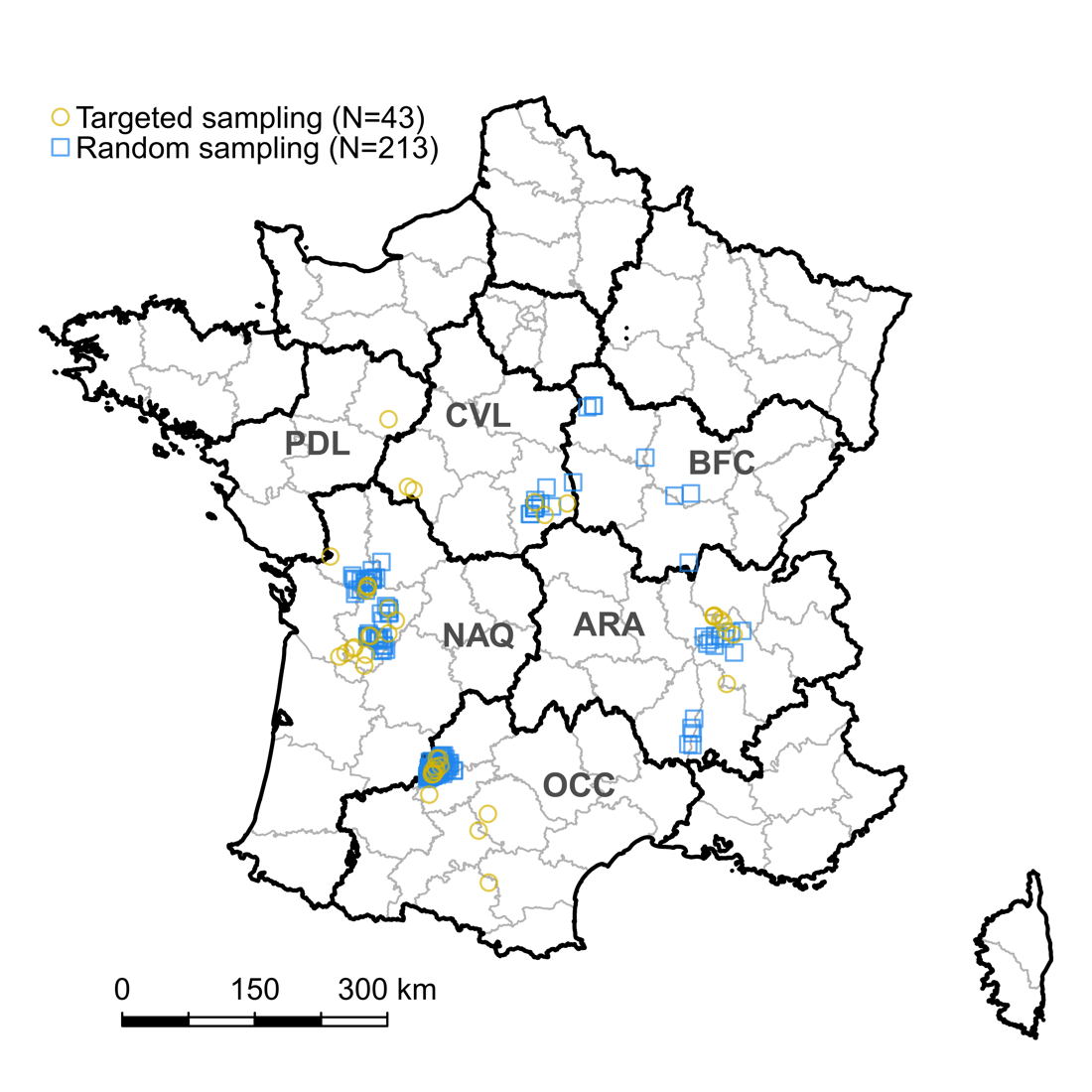


**Supplementary Figure 2:** Geographical localisation of the 256 common ragweed populations sampled for this study in six French regions. ARA, Auvergne Rhône-Alpes; BFC, Bourgogne Franche-Comté; CVL, Centre Val-de-Loire; NAQ, Nouvelle Aquitaine; OCC, Occitanie, PDL, Pays de la Loire. Map generated using R software (v4.1.0, packages rgdal, rgeos and mapplots).

**Supplementary Table 2:** common ragweed genes used as references for the normalisation of gene expression data.

| **Gene code** | **Gene** | **Primer (Forward)** | **Primer (Reverse)** |
| --- | --- | --- | --- |
| EF | Elongation factor | 5’ CGTTCATGCTGCTTTCACAAAAC | 5’ CCTCATCAGCTGCAGCCTTTGTC |
| FIL | Filamin | 5’ TTCGATGGGATTATGCCGGA | 5’ ACCGCCAAAAGAAACCATCC |
| UBQ | Cue protein, ubiquitin system component | 5’ TGAAAGAACCCTCAACCAATGTG | 5’ CACTTTGGGTTTGAATTTGATACTCT |
